# Towards human cardiac new approach methodologies (NAMs) to evaluate the combination of repolarization prolonging and shortening drugs: a pilot study

**DOI:** 10.3389/fddsv.2025.1679626

**Published:** 2025-11-10

**Authors:** Robert M. Geiger, Carlos Serna, Bhavya Bhardwaj, Tromondae K. Feaster, Ksenia Blinova

**Affiliations:** Division of Applied Regulatory Science, Office of Clinical Pharmacology, Office of Translational Sciences, Center for Drug Evaluation and Research, U.S. Food and Drug Administration, Silver Spring, MD, United States

**Keywords:** new approach methodologies (NAMs), hiPSC-CM, electrophysiology, NonclinicalStudy, complex *in vitro* model, microphysiological systems (MPS), multielectrode array (MEA), drug combination

## Abstract

**Background and Purpose:**

Nonclinical human cardiac new approach methodologies (NAMs), including human induced pluripotent stem cell-derived cardiomyocytes (hiPSC-CMs) combined with multielectrode array (MEA) represent a highly predictive *in vitro* model for identifying drug-induced cardiac liabilities of individual drugs. Here, we extend the use of an *in vitro* cardiac NAM to evaluate the safety of a drug combination including moxifloxacin, an antibiotic, and QT prolonging drug, and cobicistat a pharmacokinetic booster shown to shorten repolarization *in vitro*.

**Methods:**

To generate the *in vitro* cardiac NAM, MEA coupled with hiPSC-CMs were cultured for 7–8 days. Cells were treated with moxifloxacin and cobicistat individually or in combination and changes in electrophysiology and contractility were evaluated.

**Results:**

The combination of cobicistat and moxifloxacin resulted in a concentration-dependent shortening of the corrected field potential duration (FPDcF) relative to both vehicle control and moxifloxacin alone. This effect was observed at near clinical Cmax concentrations of cobicistat and moxifloxacin. Evaluation of local extracellular action potentials (LEAP) revealed early afterdepolarizations (EADs) with supratherapeutic concentrations of moxifloxacin which were subsequently eliminated by the addition of cobicistat at therapeutic concentrations. Finally, the Comprehensive *in vitro* Proarrhythmia Assay (CiPA) Torsades de Pointes (TdP) risk tool categorized moxifloxacin treated cells as having a high or intermediate risk probability for TdP while concomitant treatment with cobicistat resulted in a low-risk categorization.

**Conclusion:**

We conclude that cobicistat can attenuate moxifloxacin induced FPDcF prolongation at clinically relevant concentrations *in vitro*. Taken together, this work provides a foundation to evaluate drug combinations *in vitro* to aid regulatory decision-making and reduce the dependence on animal studies.

## 1 Introduction

Drug-induced ion channel block may increase the risk for Torsades de Pointes (TdP), an often-fatal arrythmia. For example, block of the human Ether-à-go-go-Related Gene (hERG) channel can result in QT prolongation, potentially leading to TdP (Yap and Camm, 2003). In contrast, late sodium channel block is shown to prevent drug induced QT prolongation and reduce the risk of TdP, suggesting that drug combinations that block both inward and outward currents may be safer than individual drug treatments that block only outward currents (Johannesen et al., 2016). However, the risk of a drug combination causing TdP prior to clinical trials is often unknown.

To better understand the clinical outcome of drugs that block multiple ion channels, the U.S. Food and Drug Administration (FDA), in collaboration with industry and academic partners, developed the Comprehensive *in vitro* Proarrhythmic Assay (CiPA) initiative. This program leverages *in silico* and *in vitro* models to better evaluate the risk of a compound causing TdP rather than exclusively relying on QT interval prolongation from animal models (Fermini et al., 2016; Sager et al., 2014). Consistent with the FDA Roadmap 2025 and FDA Modernization Act 2.0 (FDA Modernization Act, 2021; FDA, 2025) a potential alternative to traditional animal testing in regulatory evaluation is through a new approach methodology (NAM) using human induced pluripotent stem cell-derived cardiomyocytes (hiPSC-CMs) coupled with multielectrode array (MEA). This *in vitro* method has been shown to be predictive of drug-induced cardiotoxicity observed in the clinic when coupled with electrophysiology readouts such as those generated by MEA platforms (Ando et al., 2017; Blinova et al., 2018; Blinova et al., 2017; Kitaguchi et al., 2016; Millard et al., 2018). MEA measures field potentials or voltage changes that occur through a hiPSC-CM syncytium in a high-throughput manner. This *in vitro* field potential duration (FPD) shares similarities to both an action potential from an individual cell and an electrocardiogram (ECG) from cardiac tissue (Hayes et al., 2019; Yasuyuki et al., 2010). The FPD representing the time from initial depolarization represented by the sodium spike to the peak of the repolarization wave can be calculated and is a nonclinical surrogate to the QT interval in an ECG. As a result, drug induced changes observed in the FPD are shown to correlate well with changes observed in the clinical QT interval.

HiPSC-CM have the advantage of expressing multiple cardiac ion channels observed in humans. Whereas MEA have the capability to rapidly measure several cardiac excitation-contraction parameters including FPD, local extracellular action potential (LEAP), and contractility (i.e., impedance) (Du et al., 2025; Guerrelli et al., 2024; Hayes et al., 2019; Velayutham et al., 2024), all while maintaining high-throughput capabilities, allowing the simultaneous testing of multiple drug concentrations and combinations. Therefore, this model has the potential to inform dose selection of a drug combination prior to clinical trials.

The hiPSC-CM CiPA assay is routinely used to assess cardiac liability caused by individual drugs, however there is opportunity to extend their use to evaluating drug combinations. In a previous study, we evaluated the concordance of drug combinations *in vitro* (Blinova et al., 2017) relative to clinical trials (Johannesen et al., 2016). Further studies, in additional chemical space, are required to assess the use of current NAMs to predict the clinical outcome of specific drug combinations.

To evaluate this question, we identified a combination of drugs that are known to have opposing effects on cardiac repolarization (Blinova et al., 2019; FDA.gov, 2012). Moxifloxacin, an antibiotic that can cause QT interval prolongation, predominantly by hERG block, and cobicistat a pharmacokinetic booster, with multichannel blocking properties, shown to shorten repolarization in nonclinical animal models (FDA.gov, 2014). Using this model, we evaluated the electrophysiological changes (e.g., FPD) after exposing the cells to a combination of cobicistat and moxifloxacin. We show that moxifloxacin alone prolongs the Fredericia corrected field potential duration (FPDcF), but when used in combination with cobicistat, prolongation is attenuated.

## 2 Materials and methods

### 2.1 Cell culture

**FIGURE 1:**
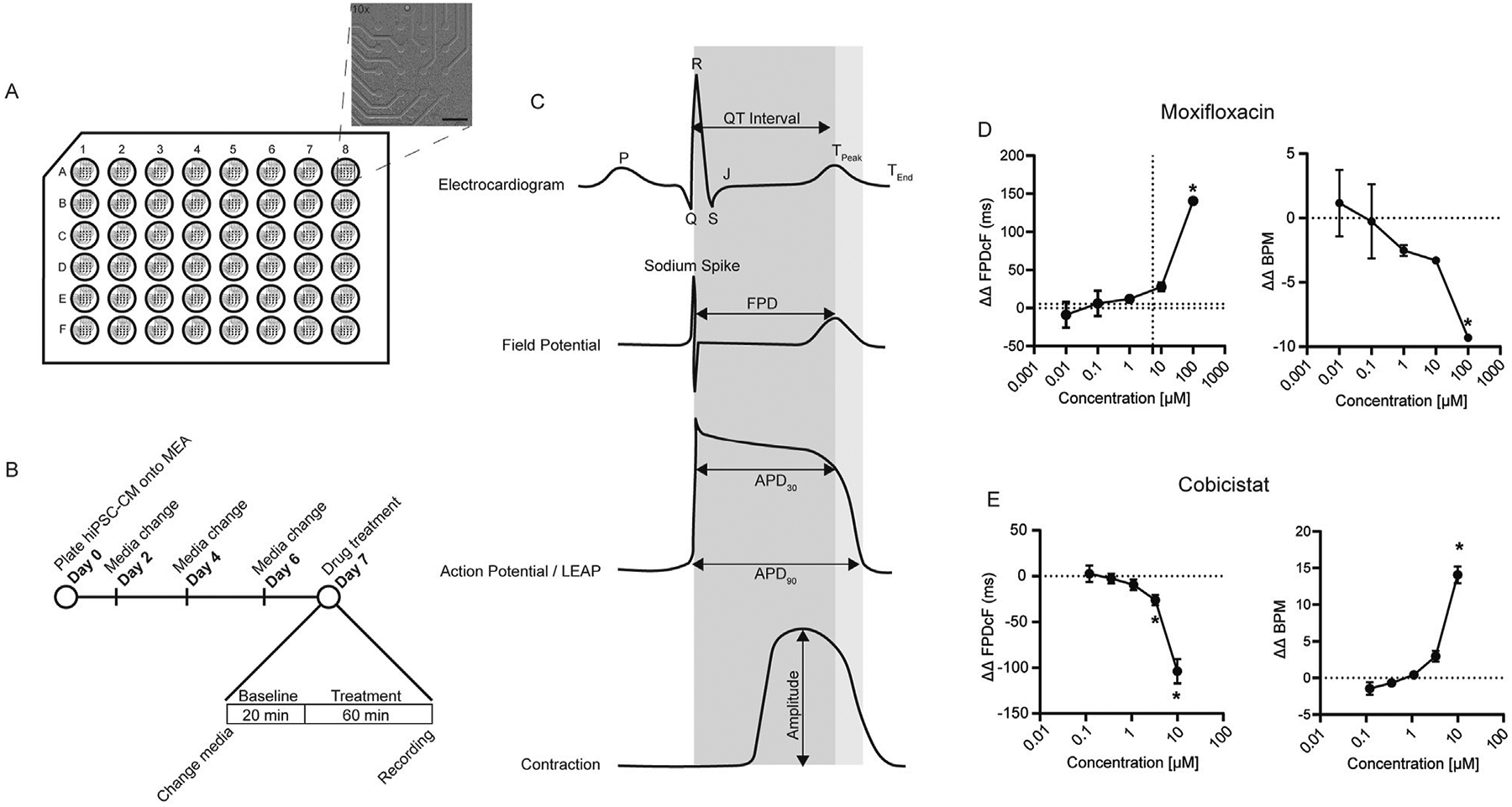
Nonclinical cardiac NAM. **(A)** Schematic of hiPSC-CMs plated on 48-well MEA plate insert indicates 16-electrode grid (region of interest). Bright field image of hiPSC-CMs covering electrodes (×10 magnification, scale bar 400 μm). **(B)** Experimental timeline and drug treatment schedule. **(C)** Example waveforms of interest demonstrating key nonclinical electrophysiological and contractile parameters relative to clinical electrocardiogram. APD, action potential duration. FPD, field potential duration. MEA, Multielectrode Array. **(D)** Moxifloxacin ΔΔ FPDcF, ΔΔ BPM, Data are mean ± SEM. n = 4 to 6 per condition, Vehicle vs. moxifloxacin (*p-value <0.05). **(E)** Cobicistat ΔΔ FPDcF, ΔΔ BPM, Data are mean ± SEM. n = 6 per condition, Vehicle vs. cobicistat (*p-value <0.05).

Human induced pluripotent stem cell-derived cardiomyocytes were obtained from Fujifilm Cellular Dynamics (FCDI) (iCell Cardiomyocytes^2^ 01434, Lot Numbers 107486, 107246, Catalog Numbers, R1017, R1059) were thawed and plated as described in the manufacturer’s protocol and as performed in our previous publication (Blinova et al., 2018). iCell Cardiomyocytes^2^ were derived from a hiPSC line that was reprogrammed from fibroblasts (donor, apparently “healthy” normal Caucasian female, <18 years old) (Feaster et al., 2021; Ma et al., 2011). In brief, 48-well multielectrode array plates (Axion BioSystems, Catalog Number, M768-tMEA-48W) were coated with 8 μL of 0.05 mg/mL fibronectin for 1 h at 37 °C. iCell Cardiomyocytes^2^ were thawed and mixed with prewarmed plating media (Fujifilm Cellular Dynamics, Catalog Number, M1001) and a volume of 8 μL at a concentration of 6.25 × 10^6^ cells/mL was plated onto the fibronectin coated plates for a total of 50,000 cells/well. Cells were allowed to adhere for 1 h to form a monolayer or syncytium on top of the electrodes. After attachment, 300 μL of maintenance media was gently added to each well. Maintenance media was changed every other day until initiation of experiments (Figure 1A).

### 2.2 MEA recordings

Recordings from hiPSC-CM multielectrode array were acquired as previously described (Tran et al., 2020). Briefly, field potentials were recorded on Days 7–8 following cell plating (Day 0). Prior to recording, cells were washed 2X with 300 μL of pre-warmed FluoroBrite^™^ DMEM (ThermoFisher, Catalog Number, A1896701). After the last wash, 270 μL FluoroBrite was added to each well. The cells were then incubated at 37 °C for approximately 1 h prior to placing the MEA plate into the Maestro Pro Platform (Axion BioSystems). The cells were allowed to equilibrate at 37 °C with 5% CO_2_ for approximately 20 min prior to measuring spontaneous baseline recordings of field potential and contractility of the syncytium (Figure 1B). A volume of 30 μL containing 10X concentration of drug in FluoroBrite^™^ DMEM (cobicistat, Caymen Chemical, Catalog Number, 23433) (moxifloxacin, Selleckchem, Catalog Number, S1465) were added to each well. The final DMSO concentration was ≤0.2% in vehicle control and drug wells. Field potential or contractility recordings containing drug or vehicle were taken after 1 h of treatment using AxIS software version 2.4.2 (Axion BioSystems). The Maestro CIPA statistics compiler tool filtered out beats with spontaneous beating outside a range of 20–90 BPM, beat periods over 6 standard deviations from the mean, a beat period CoV of <5%, or a spike amplitude of <300 μV, part of the best practice recommendations (Gintant et al., 2020; Patel et al., 2019). In addition, wells with a baseline FPDcF greater than or equal to 2 standard deviations were excluded. Thirty stable beats from each recording were selected for analysis using Axis CiPA analysis tool (version 1.2.3). To identify the presence of proarrhythmic markers, such as EADs (Early After Depolarizations) or DADs (Delayed After Depolarizations) we utilized the LEAP (local extra cellar action potentials) function of the Axion Maestro Pro. LEAP recordings to generate a waveform comparable to an action potential. LEAPs were recorded after 200 μM moxifloxacin or vehicle treatment for 1 h. Following a 5 min recording, cobicistat 1.2 μM was added to each of the drug treated wells along with vehicle to the control wells. Additional 5-min recordings were captured immediately after cobicistat addition and then every 15 min thereafter.

### 2.3 Data analysis

Field potential recordings were first processed using AxIS Navigator (Axion BioSystems, version 3.6) then analyzed with the CiPA analysis tool (Axion BioSystems, version 3.2.2) to identify the “Golden Electrode” in each well. Fredericia’s rate correction was applied to correct for differences in beat rate at baseline (FPDcF) (Fridericia, 2003). To assess contraction, a “Golden Electrode” was identified based on the largest non-biphasic beat amplitude from each well. The double delta (ΔΔ) was determined by first calculating the difference between treated wells and their corresponding baseline values (ΔΔFPDcF). These values are then compared to the vehicle control (DMSO) at the respective treatment timepoint (Blinova et al., 2019; Patel et al., 2019). The CIPA TdP risk (Model 1, Dichotomous Model) was used to predict TdP risk category as low vs. high or intermediate based on compound electrophysiological response as model predictors (Blinova et al., 2018).

### 2.4 Statistical analysis

All statistical analysis was performed with GraphPad version 8 or later. Differences among treatments are presented as a mean ± standard error of the mean. Treatment groups were compared to one another with an ANOVA with Tukey multiple comparisons test or Kruskal - Wallace test for data that were not normally distributed. Normality was evaluated with a Shapiro - Wilk test. Results were considered statistically significant if the p value was less than 0.05.

## 3 Results

### 3.1 Evaluation of moxifloxacin and cobicistat alone on cardiac electrophysiology

A human cardiac NAM composed of hiPSC-CM and MEA (Figure 1A) was used to evaluate drug effects on cardiac function *in vitro*. The field potential duration corrected for beat rate (FPDcF), local extracellular field potential (LEAP), and contractility (impedance) parameters were assessed (Figure 1C) following acute drug treatment under spontaneous (non-paced) conditions. To determine if cobicistat shortens moxifloxacin-induced prolongation, we first evaluated changes in FPDcF with moxifloxacin or cobicistat individually (Figures 1D,E; Supplementary Figure S1). As anticipated, moxifloxacin treatment resulted in significantly delayed repolarization (ΔΔ FPDcF) in a concentration-dependent manner relative to vehicle control. Moxifloxacin treatment also significantly reduced spontaneous beat rate at 100 μM (Figure 1D). Conversely, treatment with cobicistat significantly shortened repolarization duration in a concentration-dependent manner and increased beat rate at 10 μM relative to vehicle control (Figure 1E). Both moxifloxacin and cobicistat individually displayed negligible effects on the additional FPD parameters investigated (e.g., sodium spike amplitude, sodium spike slope). Herein, we quantify the acute effects of moxifloxacin and cobicistat combinations on human cardiac properties relative to vehicle and time-matched control at clinically relevant concentrations.

### 3.2 Concomitant cobicistat attenuates moxifloxacin-induced FPDcF prolongation

**FIGURE 2:**
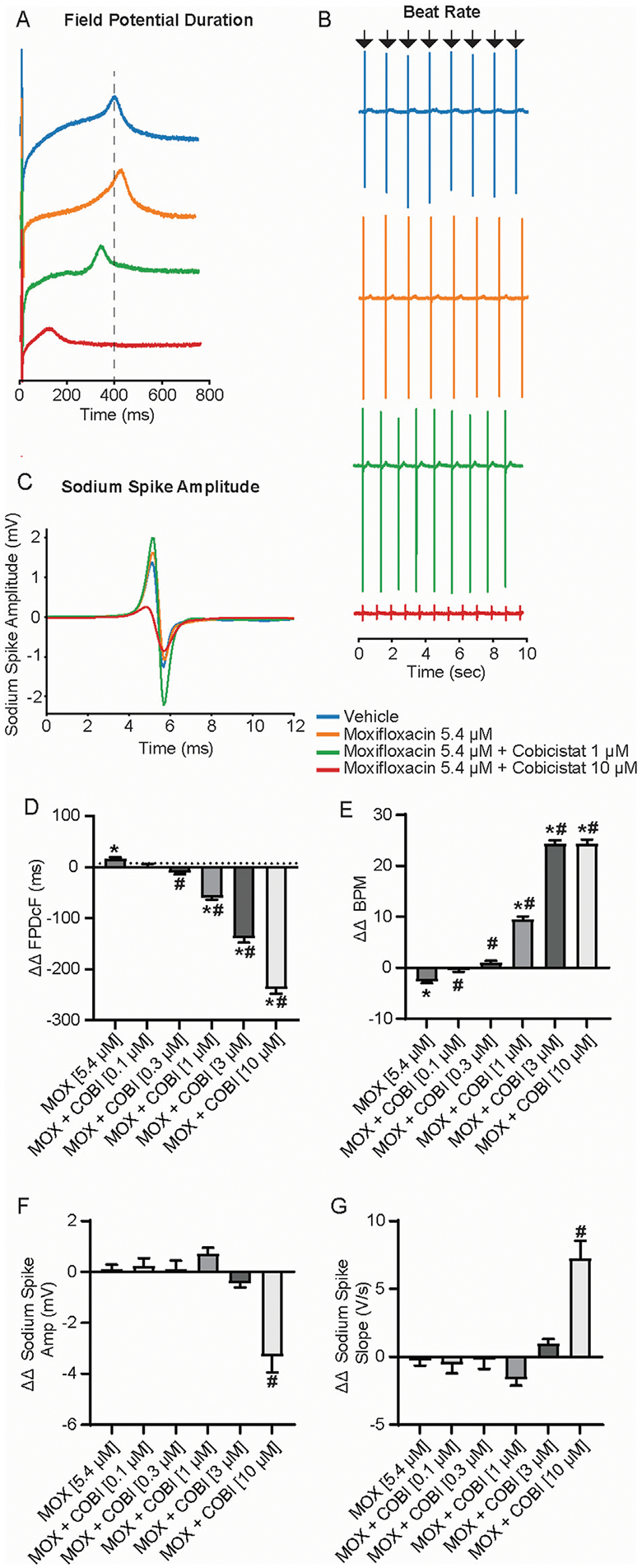
Electrophysiological Effects of Concomitant Moxifloxacin (Cmax) and Cobicistat treatment. **(A)** Representative field potential waveform from hiPSC-CMs treated with moxifloxacin (Cmax) and various concentrations of cobicistat. **(B)** Representative field potential recordings illustrating drug treatment effects on spontaneous beat rate. **(C)** Representative sodium spike waveforms demonstrating drug treatment effects on sodium spike amplitude. **(D**–**G)** Summary data graphs of field potential duration **(D)** ΔΔ FPD **(E)** ΔΔ BPM **(F)** ΔΔ Sodium Spike Amplitude, and **(G)** ΔΔ Sodium Spike Slope. Data are mean ± SEM. n = 5 to 14 per condition. Vehicle vs. treatment (*p-value <0.05); moxifloxacin 5.4 μM vs. moxifloxacin + cobicistat (^#^*p*-value <0.05).

To identify the effects of the moxifloxacin and cobicistat combination on repolarization and determine if cobicistat could attenuate moxifloxacin induced FPDcF prolongation, we evaluated the change in field potential duration. hiPSC-CM were treated with increasing concentrations of cobicistat in combination with a clinically relevant concentration of moxifloxacin 5.4 μM (Figure 2; Supplementary Table S2). Treatment of moxifloxacin alone displayed a significant increase in ΔΔ FPDcF relative to vehicle control. When combined with cobicistat, the ΔΔ FPDcF was significantly reduced in a concentration-dependent manner (Figures 2A,D) reaching significance at 0.3 μM relative to moxifloxacin alone. Addition of cobicistat also resulted in a significant increase of the spontaneous beat rate compared to moxifloxacin alone (Figures 2B,E). We next evaluated drug induced changes in sodium spike amplitude, as a reduction of the sodium current is shown to reduce the sodium spike amplitude (Harris et al., 2013). At the highest concentration tested, the combination of moxifloxacin and cobicistat induced a reduction of the sodium spike amplitude (Figures 2C,G) and a shallower sodium spike slope relative to moxifloxacin alone. Taken together, these data demonstrate that cobicistat shortens the delayed repolarization (FPDcF) induced by moxifloxacin *in vitro*.

### 3.3 Moxifloxacin prolongs field potential duration shortened by cobicistat treatment

**FIGURE 3:**
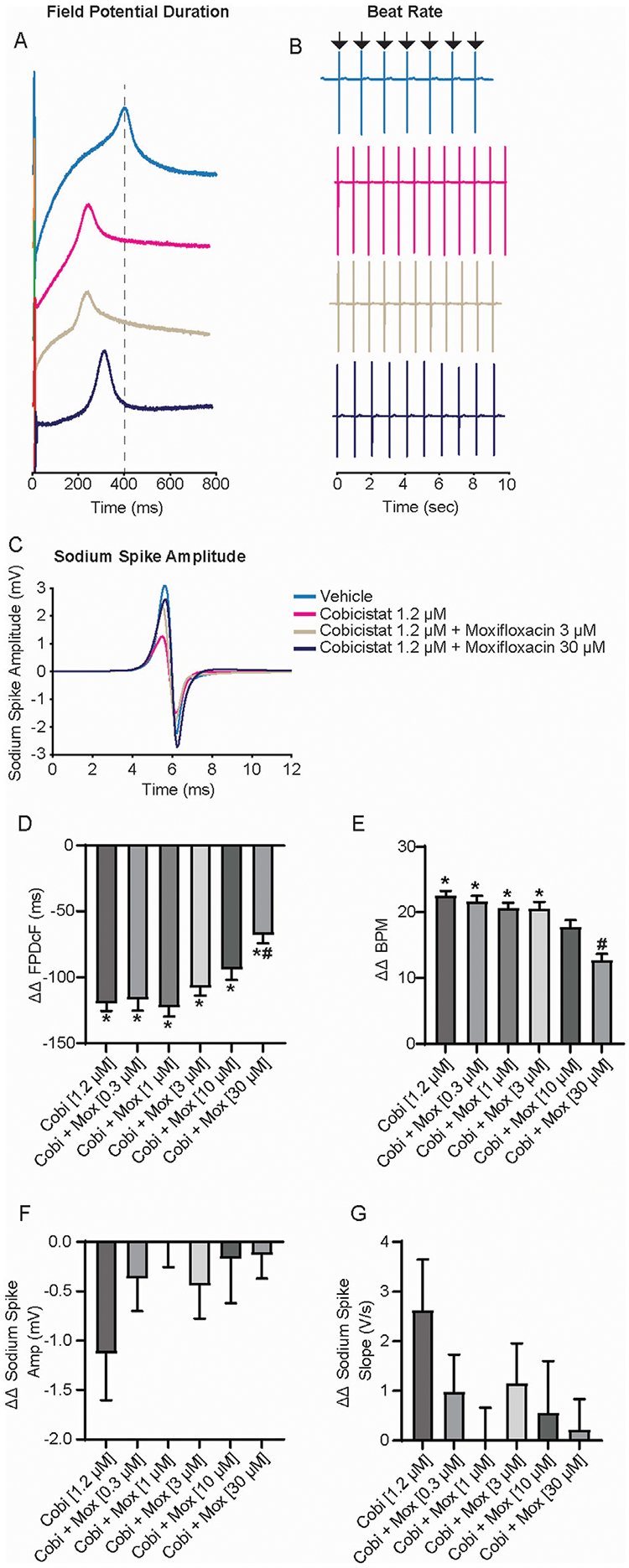
Electrophysiological Effects of Concomitant Cobicistat (Cmax) and Moxifloxacin treatment. **(A)** Representative field potential waveform from hiPSC-CMs treated with cobicistat (Cmax) and various concentrations of moxifloxacin. **(B)** Representative field potential recordings illustrating drug treatment effects on spontaneous beat rate. **(C)** Representative sodium spike waveforms demonstrating drug treatment effects on sodium spike amplitude. **(D**–**G)** Summary data graphs of field potential duration **(D)** ΔΔ FPD **(E)** ΔΔ BPM **(F)** ΔΔ Sodium Spike Amplitude, and **(G)** ΔΔ Sodium Spike Slope. Data are mean ± SEM. n = 4–6 per condition. Vehicle vs. treatment (*p-value <0.05); cobicistat 1.2 μM vs. cobicistat + moxifloxacin (^#^*p*-value <0.05).

The effects of moxifloxacin on cobicistat-induced repolarization shortening using the standard cobicistat Cmax of 1.2 μM (FDA.gov, 2012) and increasing concentrations of moxifloxacin were evaluated next (Figure 3). Despite increasing the concentration of moxifloxacin from 0.3 μM to 30 μM, the ΔΔ FPDcF remained consistently shortened when combined with cobicistat relative to vehicle control (Figures 3A,D). Furthermore, increasing moxifloxacin concentration prolonged the cobicistat-induced shortened repolarization, reaching statistical significance at 30 μM relative to cobicistat alone. Similarly, the spontaneous beat rate was significantly increased with cobicistat alone relative to vehicle control. While the addition of moxifloxacin decreased the beat rate in a concentration-dependent manner. No significant differences were observed in the sodium spike amplitude or sodium spike slope relative to vehicle control (Figures 3F,G). These results demonstrate that moxifloxacin prolongs cobicistat-induced FPDcF shortening at clinically relevant concentrations *in vitro*.

### 3.4 Evaluation of moxifloxacin and cobicistat on contraction

**FIGURE 4:**
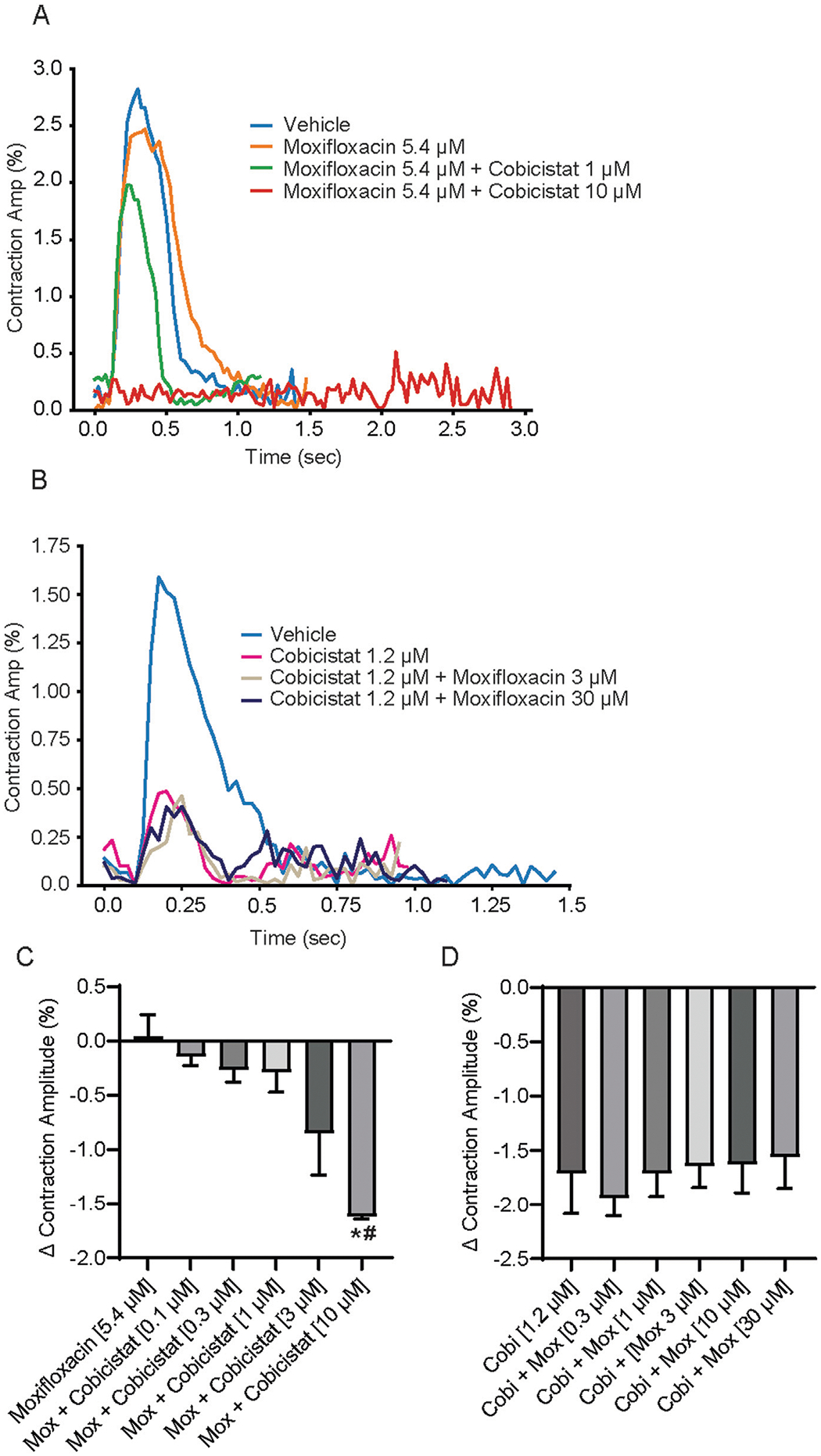
Effects of concomitant Moxifloxacin and Cobicistat treatment on hiPSC-CM Contraction Amplitude. **(A)** Representative contraction traces for hiPSC-CMs treated with moxifloxacin (Cmax) and various concentrations of cobicistat. **(B)** Summary data graphs of contraction amplitude. Data are mean ± SEM. n = 4 to 6 per condition. Vehicle vs. treatment (*p-value <0.05); moxifloxacin 5.4 μM vs. moxifloxacin + cobicistat (^#^*p*-value <0.05). **(C)** Representative contraction traces for hiPSC-CMs treated with cobicistat (Cmax) and various concentrations of moxifloxacin. **(D)** Summary data graphs of contraction amplitude. Data are mean ± SEM. n = 4 to 6 per condition. Vehicle vs. treatment (non-significant); cobicistat 1.2 μM vs. cobicistat + moxifloxacin (non-significant).

Given that cobicistat significantly shortened repolarization (ΔΔ FPDcF) and increased spontaneous beat rate both alone and in combination, we next evaluated changes in contraction amplitude. We observed that increasing cobicistat concentrations in combination with moxifloxacin (5.4 μM) caused a concentration-dependent reduction of the contraction amplitude reaching significance at 10 μM relative to vehicle control (Figures 4A,C). While increasing moxifloxacin concentrations in combination with cobicistat (1.2 μM) resulted in a non-significant decrease of the contraction amplitude relative to vehicle control (Figures 4B,D). Taken together, these data demonstrate a cobicistat-induced increase in spontaneous beat rate and a decrease in contraction amplitude consistent with L-type calcium channel blocking properties in hiPSC-CMs.

### 3.5 Cobicistat reduces moxifloxacin-induced EADs

**FIGURE 5:**
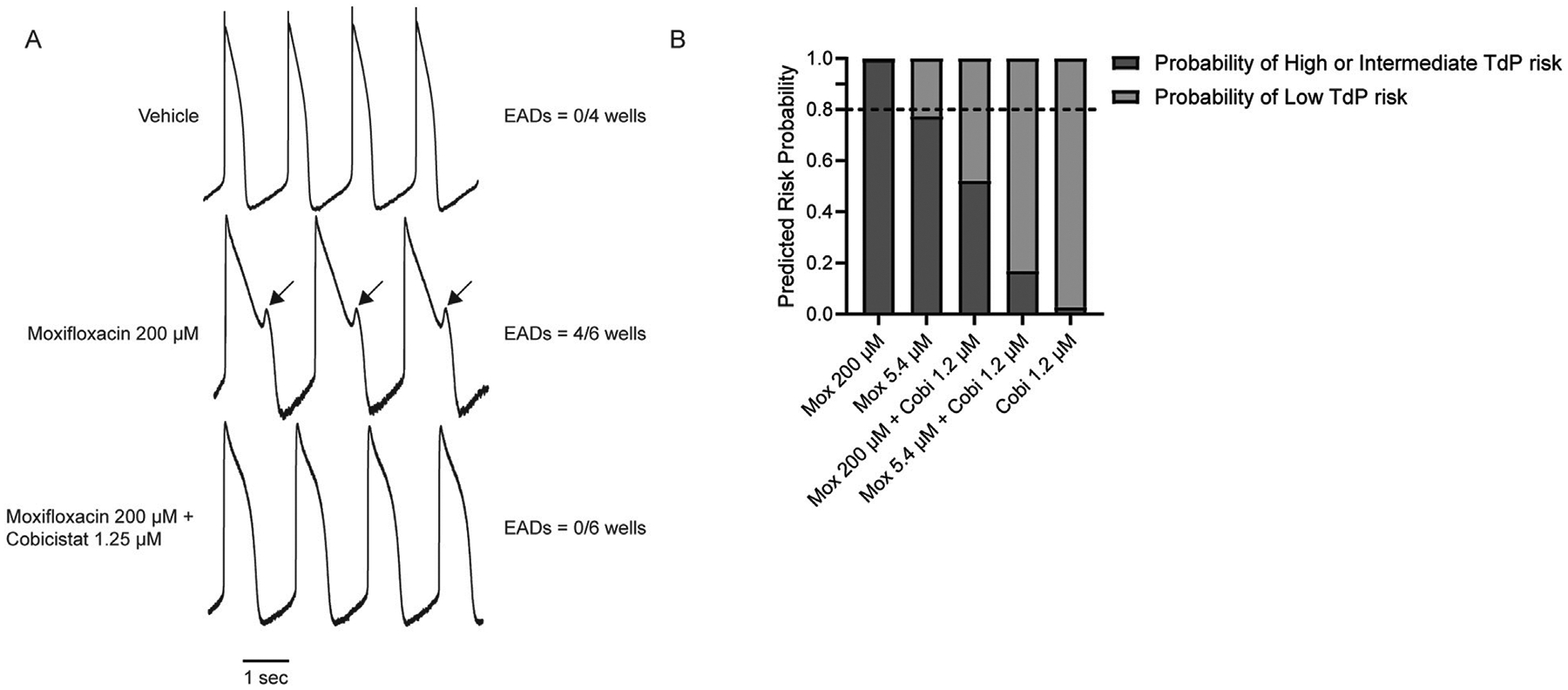
Effects of Cobicistat on Moxifloxacin-induced Proarrhythmic Markers. **(A)** Representative LEAP traces illustrating moxifloxacin-induced EAD-like events and cobicistat reversal. **(B)** TdP prediction risk categorization for concomitant Moxifloxacin and Cobicistat treatment n = 4 to 6. Horizontal dotted line represents the 0.8 threshold (Blinova et al., 2018).

While measuring the FPDcF with supratherapeutic concentrations of moxifloxacin, 200 μM, we observed type A arrhythmic-like events (i.e., biphasic repolarization wave), suggesting the presence of early after depolarizations (EADs) (Supplementary Figure S2) (Blinova et al., 2018; Blinova et al., 2017). Therefore, we used LEAP (local extracellular action potential) to verify the presence of EADs following moxifloxacin treatment and to determine if cobicistat could reduce their frequency. Indeed, we observed EADs in (4/6 wells) treated with moxifloxacin (200 μM) (Figure 5A), while subsequent treatment with 1.2 μM cobicistat reduced their frequency (0/6 wells). Next, we compared the risk of TdP with moxifloxacin alone and in combination with increasing concentrations of cobicistat to determine if cobicistat reduces moxifloxacin-induced TdP risk categorization. For this assessment, we utilized the CiPA TdP risk prediction model which uses *in vitro* compound electrophysiological responses as model predictors to categorize TdP risk as low vs. high or intermediate (Blinova et al., 2018). As expected, we observed that moxifloxacin is categorized as high or intermediate TdP risk, while concomitant treatment with cobicistat reduced this to a low TdP risk category (Figure 5B) consistent with cobicistat alone displaying low TdP risk categorization. Taken together, these data suggests that treatment with moxifloxacin and cobicistat together may display reduced cardiac liability relative to moxifloxacin alone.

## 4 Discussion

### 4.1 Cardiac NAMs respond to QT prolonging and shortening drug combination

We show that moxifloxacin prolongs the FPDcF consistent with previous reports, and we are the first to demonstrate that addition of cobicistat attenuates this prolongation *in vitro*. Furthermore, we show that the number of EADs observed with a supratherapeutic concentration of moxifloxacin can be reduced with a clinically relevant concentration of cobicistat. Likewise, the TdP categorization of moxifloxacin alone is reduced with concomitant cobicistat treatment. This study demonstrates that nonclinical cardiac NAMs including hiPSC-CMs combined with MEA can be used to evaluate electrophysiological changes induced by drug combinations.

### 4.2 Mechanisms underlying cobicistat-induced FPD shortening

Nonclinical animal studies revealed that cobicistat shortened repolarization (i.e., APD). For example, a rabbit Langendorff preparation showed decreased left ventricular function with cobicistat (1 μM) consistent with negative inotropic effects driven by calcium channel block. In addition, cobicistat demonstrated potential cardiotoxicity in dog (FDA.gov, 2014).

We used hiPSC-CMs and MEA to identify the net electrophysiological effects of moxifloxacin and cobicistat on repolarization. We observed that moxifloxacin alone prolonged the FPDcF, while cobicistat alone shortened it. However, when combined, the FPDcF was observed to be significantly reduced in the cardiac NAM. These findings suggest that multichannel block of inward currents by cobicistat can compensate for blocking of outward currents by moxifloxacin, resulting in a shortened FPDcF. These multichannel effects are demonstrated in the cardiac NAM by a simultaneous reduction of contraction amplitude and an increased spontaneous beat rate, typical of calcium channel block in hiPSC-CMs (Haoyu Zeng et al., 2020). Likewise, sodium channel block may be implicated by the reduced sodium spike amplitude of the field potential recording at higher concentrations. Future studies may explore this speculation through patch clamp to identify what ion channels are affected by the drugs.

We also investigated proarrhythmic markers with a supratherapeutic concentration of moxifloxacin to determine if they could be reduced with cobicistat. Previously, we demonstrated that 200 μM moxifloxacin can prolong the action potential duration and induce early after depolarizations (EADs) (Blinova et al., 2017). Using this same concentration, we observed EADs in cardiac NAMs when treated with 200 μM moxifloxacin. Importantly, subsequent addition of cobicistat (1.2 μM), significantly reduced their frequency suggesting that cobicistat reduces APD prolongation preventing EADs. This is consistent with other reports demonstrating that block of inward currents can reduce the QT interval and reduce the risk of arrhythmias (Johannesen et al., 2016).

Next, we applied the Comprehensive *in vitro* Proarrhythmia Assay (CiPA) Torsades de Pointes (TdP) predictivity risk categorization model to evaluate the probability of supratherapeutic moxifloxacin resulting in TdP with and without cobicistat (Blinova et al., 2018). Previously, this NAM was used to categorize TdP risk for individual drugs, we observe that the combination of cobicistat and moxifloxacin reduced the TdP risk categorization from high or intermediate to low suggesting reduced cardiac liability relative to moxifloxacin alone consistent with the proarrhythmic markers and delayed repolarization results. Taken together, these data suggests that cobicistat can reduce the potential of moxifloxacin-induced TdP and that the hiPSC-CM and MEA cardiac NAM can be used to identify the net effect of multichannel blockers on human cardiac repolarization *in vitro*.

While we demonstrate that cobicistat can mitigate moxifloxacin induced prolongation, it is important to note that cobicistat is not routinely prescribed alone or in combination with moxifloxacin. Nevertheless, the high-throughput nature of the hiPSC-CM MEA model enables simultaneous evaluation of a range of drug concentrations and combinations, thereby helping to inform a safe dose selection prior to clinical trials. As such, cardiac NAMs, where appropriate, may be useful for evaluating cardiotoxic effects of anticancer drugs as they are frequently administered in combination.

### 4.3 Potential for use in drug development

In a clinical trial conducted by the FDA, dofetilide induced QT prolongation was mitigated by inward current block using the sodium channel blockers lidocaine and mexiletine (Johannesen et al., 2016). Therefore, we used NAMs to determine if moxifloxacin induced FPD prolongation could be mitigated with cobicistat, a drug shown to shorten repolarization in nonclinical animal models (Altin et al., 2007; Haverkamp et al., 2012). We demonstrate that 5.4 μM moxifloxacin prolongs ΔΔ FPDcF by 17.02 ms and addition of 0.36 μM cobicistat significantly reduced ΔΔ FPDcF to −11.83 ms relative to vehicle. These data suggest that moxifloxacin induced QT interval prolongation may be reduced with concomitant cobicistat treatment.

Sodium channel block is shown to reduce the sodium spike amplitude of the field potential recording (Harris et al., 2013). We observed that cobicistat minimally reduced the sodium spike amplitude at low concentrations and significantly reduced the sodium spike amplitude at higher concentrations when combined with moxifloxacin suggesting a balanced multichannel block that may be beneficial. Future studies may consider evaluating the electrophysiological repolarization effects of concomitant moxifloxacin and cobicistat treatment in the clinical setting. Comparing such clinical data to the nonclinical cardiac NAM findings may enhance confidence in its ability to predict clinical outcomes of drug combinations.

### 4.4 Study limitations

Given the complexity of the system, several limitations should be considered in this study. While our results are consistent with multichannel block, it is unclear if *in vitro* drug combination effects will align with clinical findings. Moreover, future work may evaluate additional arrhythmogenic combinations to determine if the results correlate with the clinic. This may be valuable for specific patient populations (e.g., elderly) that may be vulnerable to drug-drug interactions (Permpongkosol, 2011). Using the same experimental setup described here, we have previously shown that moxifloxacin exhibits strong nonspecific binding Log P = 2.03 (Moxifloxacin, 2008; Schocken et al., 2018). Therefore, while clinically relevant concentrations were used, the free concentration may be lower than the nominal concentration. Similarly, the potential impact of nonspecific cobicistat binding on hiPSC-CM repolarization cannot be excluded. To ensure the most accurate clinical concentration ranges, we attempted to cover the standard Cmax for each drug tested as well as the Cmax observed in clinical trials (Supplementary Table S2). Future studies should consider well-exposure analysis to determine the amount of nonspecific binding in human cardiac NAMs. In addition, the cardiac NAM used here displays several features of immature cardiomyocytes including spontaneous beating, negative force-frequency, and lack post-rest potentiation (Feaster et al., 2024; Zhao et al., 2020). While previously validated for a specific context-of-use (Blinova et al., 2018), future iterations, utilizing functionally enhanced hiPSC-CMs, are expected to have enhanced predictive capabilities. Here, consistent with the standard hiPSC-CM CiPA assay, we evaluated the acute drug effects. However, chronic timepoints (e.g., days to weeks) may be of interest for specific drug combinations (e.g., hERG trafficking inhibitors).

## 5 Conclusion

This study provides a foundation for the nonclinical evaluation of FPD prolonging and shortening drug combinations on human cardiomyocyte repolarization to support safety and efficacy assessment. Here, we demonstrate several notable findings. 1) The electrophysiological effects of cobicistat alone on human cardiomyocytes *in vitro* and demonstrate significant repolarization shortening. 2) Nonclinical cardiac NAMs respond to the combination of moxifloxacin and cobicistat, at clinically relevant concentrations, by reducing moxifloxacin induced FPDcF prolongation. 3) The combination of moxifloxacin and cobicistat eliminated EAD-like events. 4) Likewise, the combination mitigated the moxifloxacin alone TdP risk categorization. Future studies may evaluate the effects of additional QT prolonging and shortening drug combinations *in vitro*. Toward that goal, we are currently evaluating various drug combinations and therapeutic modalities to assist regulatory decision making.

## Supplementary Material

Raw Data Summary

Supplementary

The Supplementary Material for this article can be found online at: https://www.frontiersin.org/articles/10.3389/fddsv.2025.1679626/full#supplementary-material

## Data Availability

The original contributions presented in the study are included in the article/Supplementary Material and at FigShare 10.6084/m9.figshare.30466295. Further inquiries can be directed to the corresponding authors.

## Abbreviations:

NAMs: New approach methodologies
MPS: Microphysiological system
TdP: Torsade de Points
CiPA: Comprehensive *in vitro* proarrhythmia assay
MEA: Multielectrode array
hiPSC-CM: Human induced pluripotent stem cell-derived cardiomyocyte
hERG: Human ether-a-go-go-related gene
APD: Action potential duration
bpm: Beats per minute
FPD: Field potential duration
EAD: Early after depolarization
LEAP: Local extracellular action potential
Cmax: Maximum clinical drug concentration in serum
