# Supplementary material for: Towards human cardiac new approach methodologies (NAMs) to evaluate the combination of repolarization prolonging and shortening drugs: a pilot study
Source: Front Drug Discov (Lausanne). Author manuscript; Available in PMC 2025 Dec 11. (PMC12690487; doi:10.3389/fddsv.2025.1679626)
Supplement: Supplementary [file NIHMS2123424-supplement-Supplementary.docx]

Supplementary Material

| Supplemental Table 1. Baseline Field Potential Electrophysiological Parameters. | | | | | |
| --- | --- | --- | --- | --- | --- |
| Parameter | | Mean | Std. Dev | SEM | N |
| Spontaneous FPD (ms) | | 336.7 | 38.10 | 2.765 | 190 |
| Beat Rate (BPM) | | 48.68 | 5.605 | 0.4066 | 190 |
| Sodium Spike Amplitude (mV) | | 6.611 | 1.371 | 0.09945 | 190 |
| Sodium Spike Slope (V/s) | -12.75 | | 3.137 | 0.2276 | 190 |

| Supplemental Table 2. Drug Pharmacokinetic Properties. | | |
| --- | --- | --- |
| Drug | Moxifloxacin | Cobicistat |
| Oral Dose, mg | 400 | 150 |
| Cmax, µM | 3.5 to 7.7 (Blinova et al., 2019; FDA) | 1.2 (FDA.gov, 2012) |
| Combination Clinical Trial (ClinicalTrials.gov) | | |


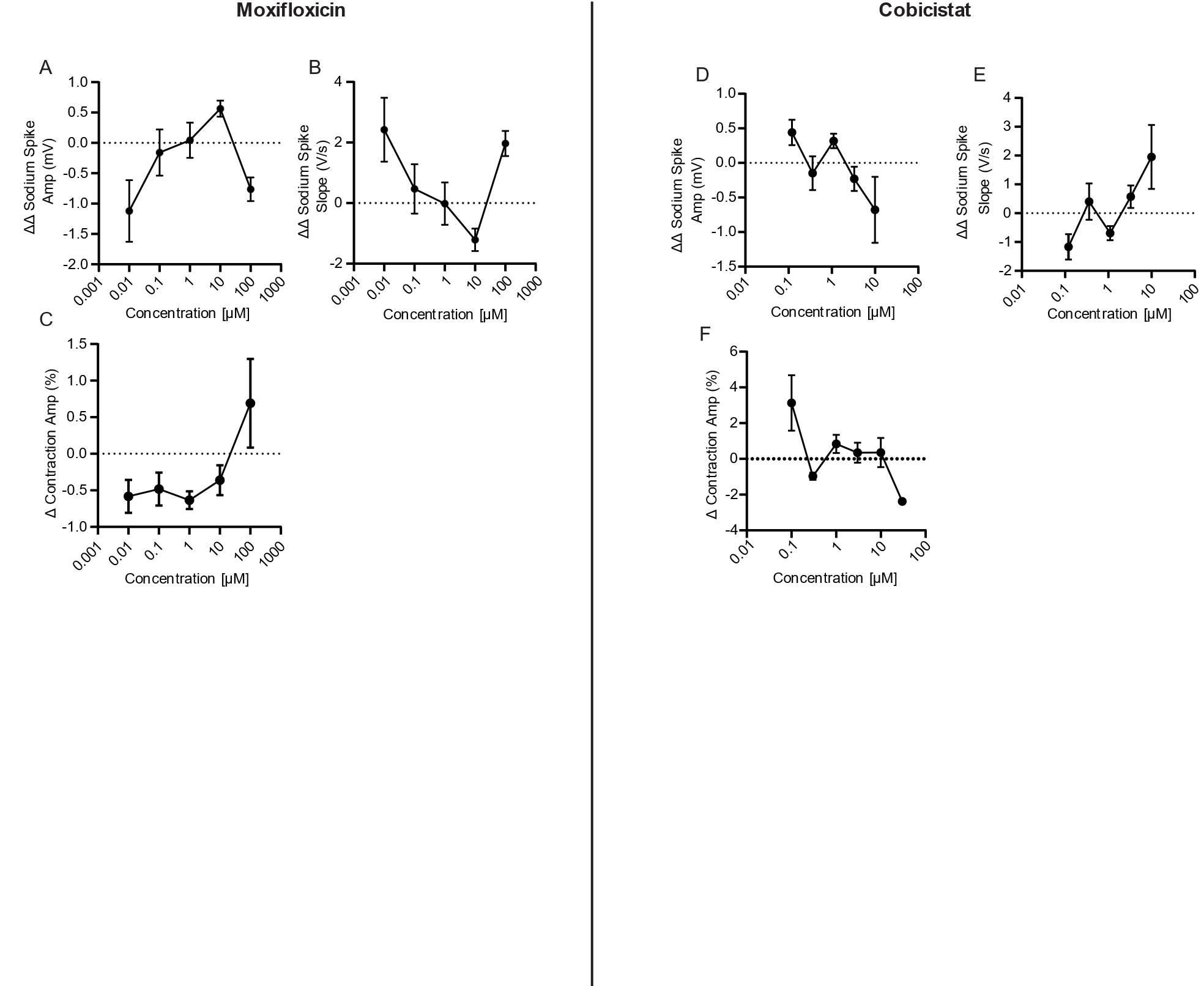


**Supplemental Figure 1.** Electrophysiological Effects of Moxifloxacin alone and Cobicistat alone. Moxifloxacin summary data graphs for **(A)** ΔΔ Sodium Spike Amplitude **(B)** ΔΔ Sodium Spike Slope **(C)** Δ Contraction Amplitude. Cobicistat summary data graphs for **(D)** ΔΔ Sodium Spike Amplitude **(E)** ΔΔ Sodium Spike Slope **(F)** Δ Contraction Amplitude. Data are mean ± SEM. n = 4 to 6 per condition. Vehicle vs treatment (**p-*value < 0.05). Horizontal dotted line represents 2 SD threshold (Blinova et al., 2017).


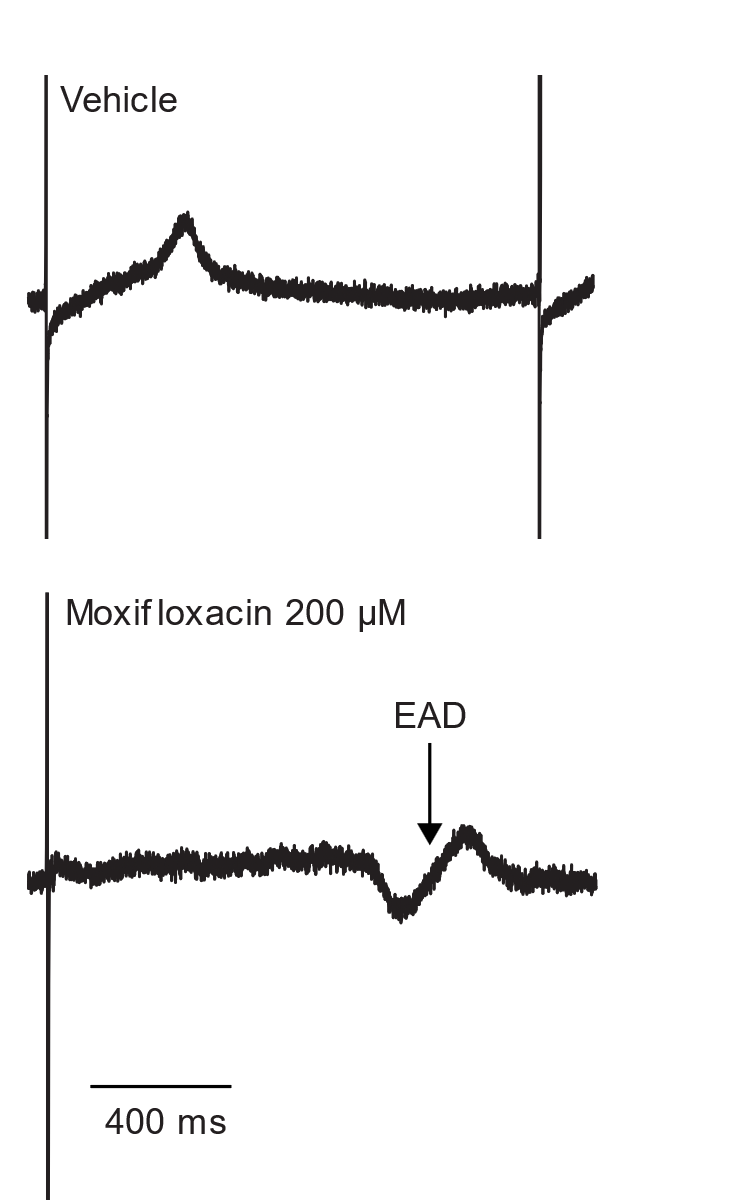


**Supplemental Figure 2.** Effects of Supratherapeutic Moxifloxacin on Field Potentials. Comparison of a field potentials recordings from hiPSC-CMs treated with a supratherapeutic concentration of moxifloxacin (200 µM) and vehicle control. Moxifloxacin-induced EAD displayed as biphasic repolarization wave as indicated by the arrow.
